# Anti-SARS-CoV-2 Immune Responses in Patients Receiving an Allogeneic Stem Cell or Organ Transplant

**DOI:** 10.3390/vaccines9070737

**Published:** 2021-07-03

**Authors:** Djordje Atanackovic, Tim Luetkens, Stephanie V. Avila, Nancy M. Hardy, Forat Lutfi, Gabriela Sanchez-Petitto, Erica Vander Mause, Nicole Glynn, Heather D. Mannuel, Hanan Alkhaldi, Kim Hankey, John Baddley, Saurabh Dahiya, Aaron P. Rapoport

**Affiliations:** 1 Transplant and Cellular Therapy Program, Department of Medicine, University of Maryland School of Medicine and Greenebaum Comprehensive Cancer Center, Baltimore, MD 21201, USA; tluetkens@som.umaryland.edu (T.L.); savila@som.umaryland.edu (S.V.A.); nhardy1@umm.edu (N.M.H.); EVanderMause@som.umaryland.edu (E.V.M.); nglynn@umm.edu (N.G.); KHankey@som.umaryland.edu (K.H.); SDahiya@som.umaryland.edu (S.D.); ARapoport@som.umaryland.edu (A.P.R.); 2 Department of Microbiology and Immunology, University of Maryland, Baltimore, MD 21201, USA; 3 University of Maryland School of Medicine and Greenebaum Comprehensive Cancer Center, Baltimore, MD 21201, USA; FLutfi@som.umaryland.edu (F.L.); gabriela.sanchez@umm.edu (G.S.-P.); HananAlkhaldi@umm.edu (H.A.); 4 Hematology/Oncology, University of Maryland Marlene and Stewart Greenebaum Comprehensive Cancer Center, Baltimore, MD 21201, USA; hmannuel@umm.edu; 5 Baltimore Veterans Affairs Medical Center, Baltimore, MD 21201, USA; 6 Division of Infectious Diseases, University of Maryland School of Medicine and Greenebaum Comprehensive Cancer Center, Baltimore, MD 21201, USA; jbaddley@ihv.umaryland.edu

**Keywords:** allogeneic stem cell transplant, COVID-19, SARS-CoV-2, antibody responses, T cells, vaccine, immunology, immunotherapy

## Abstract

Patients after autologous (autoSCT) and allogeneic stem cell transplantation (alloSCT) are at an increased risk of COVID-19-related morbidity and mortality, compounded by an immune system weakened by the underlying malignancy and prior treatments. Allogeneic transplantation, including stem cell and solid organ transplants, requires intensive immunosuppressive prophylaxis, which may further undermine the development of a protective vaccine-induced anti-viral immunity. Herein, we report on short- and long-term antiviral immune responses in two peri-stem cell transplant recipients and a third patient who received a COVID-19 vaccination after kidney transplantation. Our data indicate that: (1) patients post-alloSCT may be able to mount an anti-COVID-19 immune response; however, a sufficient time interval between transplant and exposure may be of critical importance; (2) alloSCT recipients with preexisting anti-SARS-CoV-2 immunity are at risk for losing protective humoral immunity following transplantation, particularly if the stem-cell donor lacks antiviral immunity, e.g., vaccine-derived immunity; and (3) some post-transplant patients are completely unable to build an immune response to a COVID-19 vaccine, perhaps based on the prophylactic suppression of T cell immunity.

## 1. Introduction

COVID-19 infection is caused by the Severe Acute Respiratory Syndrome Coronavirus 2 (SARS-CoV-2), and its rapid transmission around the world has resulted in a global health crisis. The SARS-CoV-2 virus contains four major structural proteins including the surface-exposed spike (S) and the internal nucleocapsid (N) proteins [1]. The S fusion protein consists of the S1 and S2 components and the virus enters cells through binding of the receptor-binding domain (RBD) within the S1 protein [2], to the angiotensin-converting enzyme-2 (ACE-2) receptor [3].

An adaptive immune response occurring in the host has the potential to control the viral infection and improve patient outcomes. Our group and others have recently shown that most patients with COVID-19 indeed develop spontaneous antibody-mediated immune responses against viral proteins [1,4,5,6] and sufficient levels of anti-SARS-CoV-2 antibodies are associated with protection from future infection [7,8]. Unfortunately, patients with hematologic malignancies, and in particular those who received stem cell transplants or novel cellular immunotherapies [9,10], show a significant degree of immunosuppression and are much less likely to quickly build a protective immune response against COVID-19 [11,12,13].

Recently, three vaccines were authorized for the prevention of COVID-19 in the United States and over half a dozen vaccines have been authorized worldwide. These vaccines have been shown to elicit antibody- and T cell-mediated antiviral immune responses conferring protection against COVID-19 [14,15,16,17,18]. However, it is still unclear if and to what extent patients who receive stem cell or solid organ transplants are able to build a vaccine-induced immune response against SARS-CoV-2. Here, we describe antiviral immune responses in two allogeneic stem cell (alloSCT) transplant patients with COVID-19 disease and one patient who received the COVID-19 vaccine following an autologous stem cell transplant (autoSCT) and solid organ transplant.

## 2. Results and Discussion

### 2.1. Case 1: Allogeneic Stem Cell Transplant Followed by COVID-19

**Figure 1:**
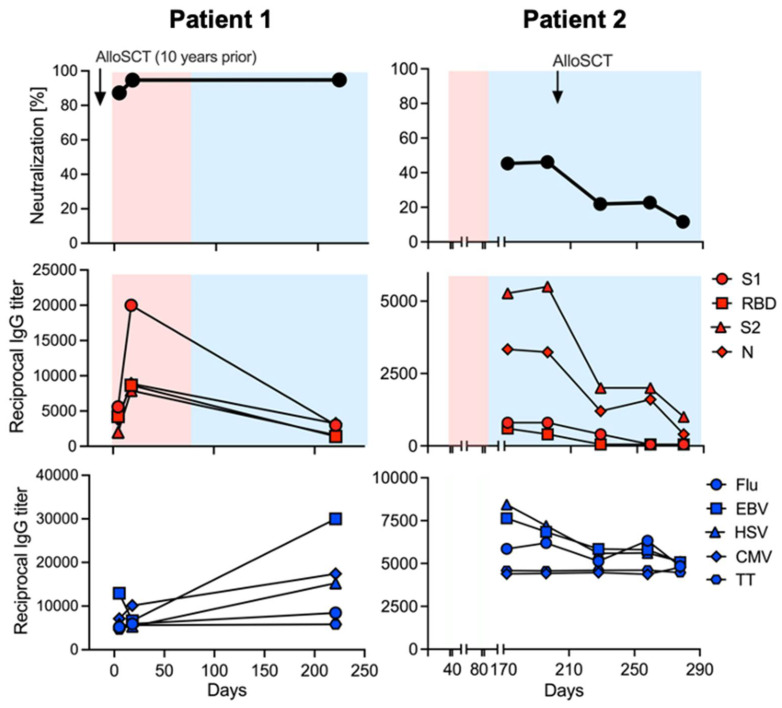
Anti-SARS-CoV antibody responses in two patients receiving allogeneic stem cell transplants. Neutralizing the activity of the patients’ serum (upper row, black symbols), antibody titers against the SARS-CoV proteins S1, RBD, S2, and N (middle row, red symbols), as well as titers against control proteins Flu, EBV, HSV, CMV, and TT (lower row, blue symbols) were measured with an ELISA. The timeframe of PCR positivity and negativity for SARS-CoV-2 are indicated by red and blue areas, respectively. The timepoint at which alloSCT was performed is indicated by a small black arrow.

A female patient in her mid-to-late 40s with a history of relapsed acute myeloid leukemia underwent a myeloablative matched unrelated allogeneic stem cell transplant 10 years prior to developing COVID-19. The patient experienced pulmonary chronic graft versus host disease (GVHD), requiring therapy for up to six years after the transplant. Of note, the patient remains in remission after the transplant and GVHD was quiescent at the time of hospital admission and in the preceding four years. The patient was intubated within two days of hospital admission for severe COVID-19. She remained on ventilator support for ~2 weeks and was treated with intravenous remdesivir. The patient was empirically treated with dexamethasone for seven days for suspected GVHD flare-up, although in retrospect she did not show evidence of GVHD. She was discharged on home oxygen after about one month in hospital. Within the first two weeks after PCR positivity, the patient developed very high IgG (Figure 1) and IgA (data not shown) antibody titers against SARS-CoV-2 proteins S1, RBD, S2, and N. Importantly, the high antibody titers led to complete viral neutralization in vitro, and clinically, the patient experienced viral clearance a few weeks later (Figure 1). Approximately 6 months later, antibody titers against viral control proteins (EBV, HSV, CMV) and vaccine-induced immune responses (Flu, TT) had remained stable or even increased but antibody titers against all four SARS-CoV-2 proteins had dramatically decreased (Figure 1). However, even the relatively lower antibody titers resulted in complete protection through viral neutralization at this later timepoint.

### 2.2. Case 2: COVID-19 Followed by Allogeneic Stem Cell Transplant

The second patient, a female in her mid-to-late 70s, had a prior history of an autologous stem cell transplant 22 years earlier for relapsed follicular lymphoma. She developed a late therapy-related AML which was treated with decitabine and venetoclax. Under treatment, she developed symptomatic COVID-19 which took about two months to resolve. After she had recovered from COVID-19 and had cleared the virus, she received an alloSCT following a reduced-intensity conditioning regimen (Figure 1) from a haploidentical related stem cell donor. She received post-transplant cyclophosphamide, tacrolimus, and mycophenolate for GVHD prophylaxis. The donor had never had COVID-19 nor any evidence of humoral anti-SARS-CoV-2 immunity at the time of donation (data not shown). Over the course of a few weeks post-transplant the patient completely lost her anti-SARS-CoV-2 immunity (Figure 1). In contrast, she maintained immunity against viral control proteins (EBV, HSV, CMV) and vaccine-induced immune responses (Flu, TT). The haploidentical donor had shown humoral immune responses against all of these microbial antigens but not SARS-CoV-2. The patient is scheduled to receive vaccination against COVID-19 when she is off of all immunosuppressive drugs.

### 2.3. Case 3: Autologous Stem Cell Transplant and Kidney Transplant Followed by COVID-19 Vaccination

**Figure 2:**
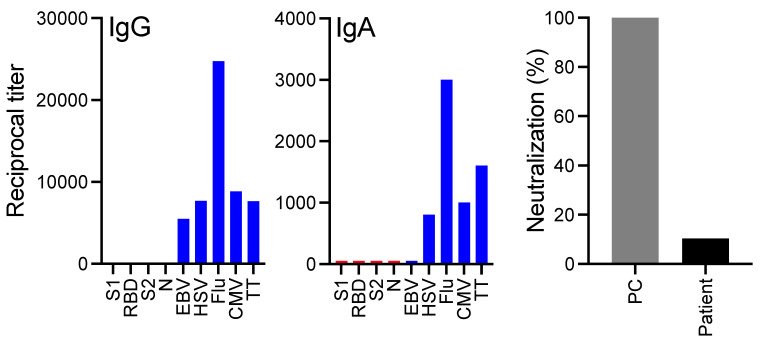
Lack of anti-SARS-CoV antibody responses to vaccination in a patient post kidney transplant. IgG and IgA antibody titers against the SARS-CoV proteins S1, RBD, S2, and N (red bars) and titers against control proteins Flu, EBV, HSV, CMV, and TT (blue bars) were measured by an ELISA. The neutralizing activity of the patient’s serum and a positive control (PC) are shown on the right.

The third patient is in her mid-60s and was originally diagnosed with light chain deposition disease (LCDD) in 2006. She received multiple types and lines of treatments, including the proteasome inhibitor bortezomib and the immunomodulatory agent lenalidomide. In 2017, her kidney function worsened because of the underlying plasma cell dyscrasia, and she was started on dialysis. At that time, treatment with the anti-CD38 antibody daratumumab was initiated and she achieved a very good partial response. In July of 2017, she received high-dose melphalan chemotherapy followed by an autoSCT. In September of 2018, the patient received a kidney transplant and started immunosuppressive therapy with tacrolimus and mycophenolic acid. She was also intermittently treated with steroids and remained on dual immunosuppression when she received two doses of the FDA-approved mRNA-1273 COVID-19 vaccine. At that time, her LCDD was in complete remission and she had normal total IgG, IgM, and IgA serum levels and normal CD4^+^ and CD8^+^ T cell counts. Unfortunately, two weeks after receiving the second dose of the vaccine, while serum antibody titers against viral control proteins (EBV, HSV, CMV) and vaccine-induced antibody levels (Flu, TT) were comparably high, IgG and IgA titers against SARS-CoV-2 proteins S1, RBD, S2, and N were non-existent. As a result, her serum did not show evidence of any SARS-CoV-2 neutralizing activity (Figure 2).

We describe here the immune responses and clinical courses of three patients with SARS-CoV-2 infection or vaccination in the context of alloSCT or solid organ transplant. The first case demonstrates that patients post alloSCT may ultimately be able to mount a strong, protective, and durable antiviral immune response; however, a sufficient time interval between transplant and exposure to the viral antigen may be of critical importance. As a consequence, clinical studies should investigate the impact of vaccination timing on the establishment of anti-SARS-CoV-2 immune responses to determine the most reasonable vaccine administration schedule for this particular patient population. Additionally, serial measurements of antibody titers will be necessary to determine the durability of viral immune responses in this severely immunocompromised group.

The second case illustrates that pre-existing SARS-CoV-2 immunity may be lost following alloSCT, particularly if the stem-cell donor shows no anti-SARS-CoV-2 immunity due to the conditioning regimen, post-transplant immunosuppression use, and the establishment of the donor-derived immune system. At the same time, this case illustrates that preexisting donor immunity is effectively transferred to the recipient following alloSCT. Plausibly, this case demonstrates how donor vaccination prior to stem cell donation might confer protection from COVID-19 to patients undergoing alloSCT. Future clinical study of anti- SARS-CoV-2 immunity in recipients of immunized donors is warranted.

The last case illustrates that there is a subgroup of post-transplant patients who are completely unable to build an immune response to a COVID-19 vaccine. Patients like our third case, where the solid organ transplant was performed relatively recently, are probably more likely to be affected by this unresponsive immunity. The fact that our patient had normal immunoglobulin levels and normal to high antibody titers to other microbial antigens, raises the possibility that it is not primarily a compromised humoral immune system that undermines a vaccine-induced immune protection but that the prophylactic suppression of T cell immunity (e.g., with calcineurin inhibitors) in these patients leads to a lack of T cell help for naïve COVID-19-specific B cells, resulting in an inability to mount a high-titered and durable antiviral antibody response. Furthermore, mycophenolic acid, through dual suppression of both B cell and T cell proliferation, limits both cell-mediated immune responses and antibody formation, with predictable deleterious effects on vaccine efficacy. Careful investigation of SARS-CoV-2 vaccination including a detailed and standardized assessment of immune responses, and possibly the use of additional “booster” vaccinations, in this complex group of immunocompromised patients [19,20], is warranted to optimize their response to the vaccine. Optimal timing of vaccination in allogeneic stem cell transplant needs to be defined. From a GVHD risk standpoint, inactivated vaccines have generally shown low risks and have not caused or worsened GVHD; thus, inactivated COVID-19 vaccines can be started three to six months after transplant.

## 3. Methods

### 3.1. Patients and Samples

We collected 40 mL of heparinized blood from three patients at the University of Maryland Medical Center between June of 2020 and February of 2021. Informed consent was obtained and blood samples were collected under IRB HP-00091425m, IRB HP-00057785, and IRB HP-00018133, respectively. Plasma was extracted from peripheral blood samples after centrifugation at 400× *g* for 10 min and frozen immediately at −80 °C.

### 3.2. Enzyme-Linked Immunosorbent Assay (ELISA)

Serum antibody responses against four recombinant, full-length SARS-CoV-2 proteins expressed in human 293 cells (Acro Biosystems, Newark, DE, USA) and viral control proteins Epstein-Barr Virus Glycoprotein gp350 (EBV), Human Cytomegalovirus Glycoprotein B (CMV), Influenza A H1N1 Nucleoprotein (Flu; Sino Biological, Chesterbrook, PA, USA), Herpes Simplex Virus 1 (HSV; Cambridge, UK), and tetanus toxoid (TT; Boehringer) were determined by enzyme-linked immunosorbent assay (ELISA) as previously described [21]. For the calculation of titers, regression analyses were performed for the linear segment of serum titration curves for positive samples and pooled sera from five healthy donors. Titers were defined as the dilution at the intersection of both regression lines.

### 3.3. SARS-CoV-2 Neutralization Assay

Neutralizing activity of patient sera was assessed using the cPass Neutralization Antibody Detection Kit (GenScript Biotech, Piscataway, NJ, USA) according to the manufacturer’s instructions. The kit detects circulating neutralizing antibodies against SARS-CoV-2 that block the interaction between the RBD of the viral spike glycoprotein with the ACE-2 cell surface receptor.
